# Contribution of σ^70^ and σ^N^ Factors to Expression of Class II *pilE* in Neisseria meningitidis

**DOI:** 10.1128/JB.00170-19

**Published:** 2019-09-20

**Authors:** Mariya Lobanovska, Christoph M. Tang, Rachel M. Exley

**Affiliations:** a Sir William Dunn School of Pathology, University of Oxford, Oxford, United Kingdom; Michigan State University

**Keywords:** *Neisseria meningitidis*, pilin, sigma factors, transcriptional regulation, type four pili

## Abstract

Neisseria meningitidis expresses multicomponent organelles called type four pili (Tfp), which are key virulence factors required for attachment to human cells during carriage and disease. Pilin (PilE) is the main component of Tfp, and N. meningitidis isolates either have a class I *pilE* locus and express pilins that undergo antigenic variation or have a class II *pilE* locus and express invariant pilins. The transcriptional regulation of class I *pilE* has been studied in both N. meningitidis and Neisseria gonorrhoeae, while the control of expression of class II *pilE* has been elucidated in the nonpathogenic species Neisseria elongata. However, the factors that govern the regulation of the class II *pilE* gene in N. meningitidis are not known. In this work, we have bioinformatically and experimentally identified the class II *pilE* promoter. We confirmed the presence of conserved σ^70^ and σ^N^-dependent promoters upstream of *pilE* in a collection of meningococcal genomes and demonstrated that class II *pilE* expression initiates from the σ^70^ family-dependent promoter. By deletion or overexpression of sigma factors, we showed that σ^N^, σ^H^, and σ^E^ do not affect class II pilin expression. These findings are consistent with a role of the housekeeping σ^D^ in expression of this important component of Tfp. Taken together, our data indicate that the σ-dependent network responsible for the expression of class II *pilE* has been selected to maintain *pilE* expression, consistent with the essential roles of Tfp in colonization and pathogenesis.

**IMPORTANCE** The type four pilus (Tfp) of Neisseria meningitidis contributes to fundamental processes such as adhesion, transformation, and disease pathology. Meningococci express one of two distinct classes of Tfp (class I or class II), which can be distinguished antigenically or by the major subunit (*pilE*) locus and its genetic context. The factors that govern transcription of the class II *pilE* gene are not known, even though it is present in isolates that cause epidemic disease. Here we show that the transcription of class II *pilE* is maintained throughout growth and under different stress conditions and is driven by a σ^70^-dependent promoter. This is distinct from Tfp regulation in nonpathogenic *Neisseria* spp. and may confer an advantage during host-cell interaction and infection.

## Abstract (precis)

The type four pilus (Tfp) of Neisseria meningitidis contributes to fundamental processes such as adhesion, transformation, and disease pathology. Meningococci express one of two distinct classes of Tfp (class I or class II), which can be distinguished antigenically or by the major subunit (*pilE*) locus and its genetic context. The factors that govern transcription of the class II *pilE* gene are not known, even though it is present in isolates that cause epidemic disease. Here we show that the transcription of class II *pilE* is maintained throughout growth and under different stress conditions and is driven by a σ^70^-dependent promoter. This is distinct from Tfp regulation in nonpathogenic *Neisseria* spp. and may confer an advantage during host-cell interaction and infection.

## INTRODUCTION

Neisseria meningitidis is a human-specific, Gram-negative bacterium that is a leading cause of meningitis and septicemia worldwide (1). Despite its ability to cause invasive disease, N. meningitidis colonizes the human nasopharynx, and it is carried asymptomatically by approximately 10% of the population (2). The capsular polysaccharide forms the basis of meningococcal classification into 12 serogroups, and meningococci can be further classified into clonal complexes based on nucleotide sequence differences in housekeeping genes (3). Certain clonal complexes, such as cc-11, have a marked propensity to cause disease and are referred to as hyperinvasive lineages (4).

Meningococci express type four pili (Tfp), which play key roles during meningococcal carriage and disease. During colonization, Tfp meditate the initial adherence of N. meningitidis to epithelial cells (5). Progression to systemic disease and dissemination from the nasopharynx are proposed to be triggered by the detachment of a small number of bacteria from microcolonies on the epithelial surface following changes in Tfp expression (6). Subsequently Tfp mediate formation of microcolonies on endothelial cells, providing resistance against shear stress in the circulation (7), as well as reorganization of host cell components, leading to translocation of N. meningitidis into the cerebrospinal fluid (8). In addition, Tfp are required for twitching motility and competence for DNA uptake that allows horizontal gene transfer between bacteria (9, 10).

The major component of Tfp is the pilin protein PilE. N. meningitidis isolates express one of two distinct classes of the type four pilin subunit: either a class I pilin, which undergoes high-frequency antigenic variation, or an invariant class II pilin (11). Based on genome analysis, meningococcal isolates have either a class I or a class II *pilE* locus but not both, and a number of features distinguish these loci (12, 13). For example, class I *pilE* loci are flanked by sequences that enable intrastrain pilin variation, namely, a G-quadruplex-forming (G4) sequence upstream of the *pilE* open reading frame (ORF) and a downstream Sma/Cla sequence. These features facilitate nonreciprocal homologous recombination between *pilE* and silent *pilS* cassettes located immediately downstream of *pilE* (14–17). Isolates that express invariant pilins harbor a class II *pilE* locus. This is situated at a different chromosomal site, and therefore, although *pilS* cassettes are present, they are not adjacent to *pilE*. Furthermore, the G4 and Sma/Cla elements are lacking from the regions adjacent to the class II *pilE* locus (12, 13, 18). Of note, strains with class II *pilE* mostly belong to hyperinvasive lineages that are responsible for epidemic disease in sub-Saharan Africa and for worldwide outbreaks (13, 19, 20).

A number of studies have investigated the transcriptional regulation of *pilE* in pathogenic and nonpathogenic *Neisseria* spp. (21–24). These have shown that the number, arrangement, and activity of *pilE* promoters vary between species and strains. For example, the class I *pilE* promoter in N. meningitidis strain MC58 includes both −10/−35 and −12/−24 sequences, but the −12/−24 sequence does not play a role in pilin transcription (21). The same is true in the related species Neisseria gonorrhoeae, in which the *pilE* promoter is similar to the meningococcal class I *pilE* promoter (25). In contrast, the *pilE* promoter of the nonpathogenic species Neisseria elongata lacks −10/−35 sequences, and transcription initiates from a −12/−24 sequence (23).

All bacterial promoters are recognized by sigma (σ) factors. These can be divided into two groups: (i) the σ^70^ family, which are structurally related factors that recognize −10/−35 sequences, and (ii) σ^N^, which recognizes a consensus −12/−24 sequence and has a noncanonical mechanism of transcription initiation, requiring interaction with an AAA+ ATPase activator protein (26, 27). The meningococcal genome contains four genes encoding potential σ factors: three σ^70^ factors (σ^D^, σ^E^, and σ^H^) and σ^N^. σ^D^ is considered the housekeeping σ factor, σ^E^ is autoregulated by an anti-sigma factor and regulates 11 genes (28), and σ^H^ is essential but as yet has no defined role in the meningococcus, although it has been linked with the response to heat shock in gonococci (29, 30). σ^N^ has been reported to be nonfunctional based on analysis of isolates in which the *rpoN* gene is similar to the *rpoN*-like sequence (RLS) of N. gonorrhoeae (25). The gonococcal RLS has a frameshift mutation that results in loss of the domains required for DNA binding, namely, the helix-turn-helix (HTH) motif and the RpoN box which are required to bind to the −12 and −24 sequences, respectively (23, 25). More recently, genome sequence analysis has demonstrated that different meningococcal isolates encode distinct forms of σ^N^, some of which lack both of the key DNA binding domains and others of which lack only the HTH motif (23). Interestingly, in N. elongata *rpoN* encodes a protein with both DNA binding motifs and which activates *pilE* transcription (23).

The factors that govern transcription of meningococcal class II *pilE* have not been studied previously. In this work, we characterized the *pilE* promoter in an N. meningitidis isolate expressing class II pilin (31). Through bioinformatic analysis of whole-genome sequences (WGS), we found that the class II *pilE* promoter region is highly conserved. The region comprises consensus σ^70^-dependent and σ^N^-dependent promoter sequences. We show that class II *pilE* is expressed throughout bacterial growth and is transcribed from the σ^70^-dependent promoter. Our data showed that neither deletion of meningococcal *rpoN* nor overexpression of σ^N^ had any effect on pilin levels. Furthermore, altered expression of two other meningococcal σ factors, σ^E^ and σ^H^, did not alter pilin levels, suggesting that class II pilin-expressing N. meningitidis strains have selectively retained housekeeping σ^D^–dependent pilin transcription, allowing its constitutive expression.

## RESULTS

### Class II *pilE* promoter regions are conserved and distinct from class I *pilE* promoters.

**FIG 1:**
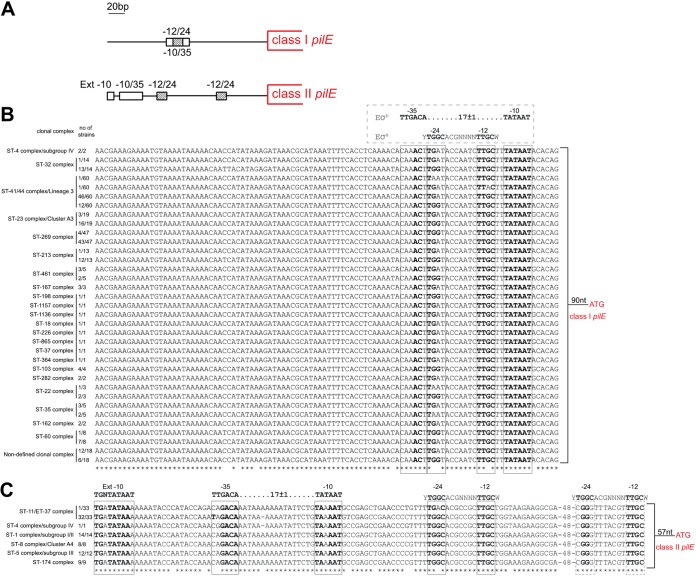
Analysis of class I and class II *pilE* promoter regions in N. meningitidis. (A) Schematic representation of the class I and class II *pilE* promoter regions, based on N. meningitidis MC58 and 8013 (class I *pilE*) and FAM18 (class II *pilE*). An extended −10 and −10/−35 sequences are shown as open boxes, and the −12/−24 sequences are shown as striped boxes. (B and C) Clustal Omega alignment of the region upstream of *pilE* in isolates with class I (B) and class II (C) *pilE*. The clonal complex and the number of strains with the indicated sequences are shown. Sequences corresponding to −10/−35 and −12/−24 elements are boxed. E. coli consensus sequences (Eσ^D^ −10/−35 and Eσ^N^ −12/−24) are shown above the alignments. Asterisks indicate positions which have a single, fully conserved residue. Nucleotides in bold are identical to the consensus sequences.

The sequence and organization of the *pilE* promoter in N. meningitidis have been described previously (12, 23, 24). Rendon et al. analyzed 64 meningococcal *pilE* promoter regions and classified them into three groups with different compositions of σ^70^-dependent and σ^N^-dependent sequences (23). Independent analysis of genomes of meningococcal isolates expressing class I Tfp (8013 and MC58), and class II Tfp (FAM18) has shown that the compositions and sequences of the promoters of class I and class II *pilE* genes are distinct (12) (Fig. 1A). To determine whether this observation holds true for other strains, we analyzed *pilE* promoters in a collection of 290 meningococcal genomes. The collection included strains belonging to 20 different clonal complexes, isolated from 30 different countries, and comprised 213 isolates with class I *pilE* and 77 isolates with class II *pilE* (13). The sequence of the *pilE* promoter region was extracted from the PubMLST database using the full-length pilin-coding sequence from either 8013 (class I) or FAM18 (class II) as the query sequence and extracting 500 bp of 5′ flanking sequence. The nucleotide sequences of *pilE* and the promoter regions were aligned using Clustal Omega, and comparison with previously described meningococcal *pilE* promoter sequences (12, 21) and/or consensus sequences (32, 33) was used to annotate extended −10, −10/−35, and −12/−24 sequences. Our analysis revealed that the σ^70^- and σ^N^-dependent promoter elements reported previously were present in all 213 class I *pilE*-containing isolates and that the relative positions of the −10/−35 and −12/−24 sequences were conserved (Fig. 1B). The sequences of the −10 and −35 elements were identical across all 213 isolates, and the −10 sequence was an exact match with the bacterial consensus −10 sequence (TATAAT). The −35 sequence, however, contained only two out of six consensus nucleotides (caaACt, compared to the consensus TTGACA), in line with previous reports that −35 sequences are not conserved in meningococcal genomes (21, 34). The σ^N^-dependent promoter was located between the −35 and −10 sequences in all isolates but showed some sequence variation, and 138 of 213 strains contained at least one nonconsensus nucleotide in the −24 sequence (Fig. 1B), with one isolate, belonging to the ST-41/44 complex, having an additional change in the −12 region.

Similarly, the σ^70^- and σ^N^-dependent *pilE* promoters described in FAM18 (12, 23) were identified in all 77 genomes of strains with class II *pilE* (Fig. 1C). The extended −10 sequence was identical across all 77 isolates examined, and the sequence and spacing of the −10/−35 sequences of the σ^70^-dependent promoter were identical in 43 of the 77 isolates. Interestingly, in 1/1 cc-4 isolate and 32/33 cc-11 isolates the −10 and −35 elements were separated by 16 rather than 17 nucleotides, due to deletion of single adenine from a poly(A_7_) tract, although this still conforms to consensus σ^70^-dependent promoter spacing (35). Unlike the putative −35 sequence in the class I *pilE* promoters, the −35 sequence in the class II *pilE* promoters had at least four out of six nucleotides conserved (C/T-A/G-GACA [consensus, TTGACA]).

Two putative σ^N^-dependent promoters were identified in all 77 isolates with class II *pilE*. These were previously reported in the *pilE* promoter region of FAM18 (12), and, similar to the published findings, one of the −12/−24 promoters had nonconsensus nucleotides in the −24 sequence (CGGG instead of TGGC), including nucleotide changes that have been shown experimentally to impair the activity of the σ^N^ promoter (32). The sequence of the second σ^N^-dependent promoter was conserved and exhibited 100% nucleotide sequence identity to the −12/−24 consensus in all but one of the 77 isolates (Fig. 1C). This sequence conservation and lack of overlap with the σ^70^ binding site raised the possibility that the −12/−24 sequence is implicated in class II *pilE* regulation. Since the −12/−24 sequence requires σ^N^ for transcription initiation, we next examined the presence and sequence of *rpoN*, which encodes σ^N^.

### Meningococcal isolates contain atypical *rpoN* sequences.

**FIG 2:**
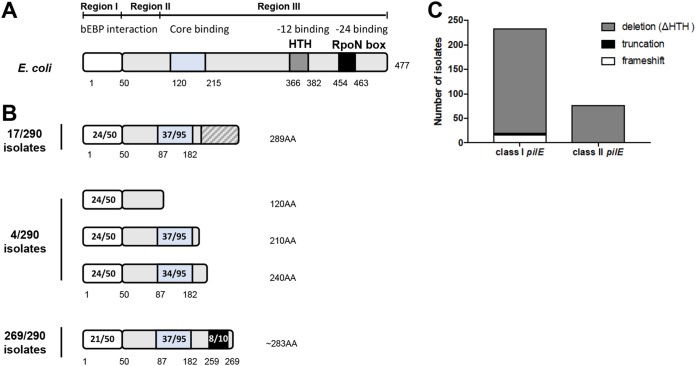
Schematic diagrams of σ^N^ proteins in E. coli and N. meningitidis. (A) Schematic representation of the E. coli σ^N^ domain organization. Three regions have been defined: region I mediates interactions with activator proteins (bEBPs), region II is a linker with variable sequence, and region III comprises an RNAP core binding domain and two DNA binding domains, the HTH motif and RpoN box. (B) Schematic of the deduced domain organization of σ^N^ in N. meningitidis. Analysis of genomes of 290 meningococcal isolates revealed 34 different peptide sequences. Putative domains were identified based on EMBOSS Water pairwise sequence alignment with E. coli σ^N^. Meningococcal σ^N^ was classified into three groups. σ^N^ proteins present in 17/290 isolates and 4/290 isolates harbor frameshift mutations or are truncated, respectively, and therefore lack any DNA binding domains. In the majority of isolates (269/290, 93%) σ^N^ lacks the HTH domain but retains an in-frame RpoN box. Amino acid numbers are shown. Numbers in the boxes indicate the numbers of identical amino acids compared to E. coli σ^N^. (C) Distribution of putative σ^N^ types (frameshift, truncation, or deletion) in isolates with class I or class II *pilE*.

The *rpoN* gene in Escherichia coli (1,434 bp) is present in a single copy and encodes a protein of 477 amino acids (aa) with three domains. Region I is the activator binding domain, region II is a variable, nonessential region involved in DNA melting, and region III interacts with RNA polymerase (RNAP) and is implicated in promoter recognition and binding (26) (Fig. 2A). Region III contains two DNA binding/recognition motifs: an HTH motif (36) and the RpoN box (ARRTVAKYRE) (27). In N. gonorrhoeae the RLS is predicted to encode a protein of 277 aa which lacks the HTH motif and RpoN box; hence, any protein product is likely to be inactive (23, 25). In N. meningitidis 8013, the *rpoN* ORF encodes a potential 289-aa protein also without any recognizable HTH motif and which lacks the RpoN box due to a frameshift mutation (25, 37). However, more recently several N. meningitidis isolates were reported to harbor an *rpoN* sequence that encodes a protein which retains the RpoN box at the C terminus (23). We therefore investigated the presence and sequence conservation of *rpoN* in the collection of 290 N. meningitidis isolates.

The *rpoN* sequence from FAM18 (NEIS0212 allele 1 in PubMLST) was used as the query to extract sequences using BLASTn in PubMLST. We identified *rpoN* homologues in all 290 genomes. In total, there were 44 different alleles among the 290 isolates (see Table S1 and Fig. S1 in the supplemental material). The *rpoN* sequences were translated using EMBOSS Transeq, and predicted amino acid sequences were aligned using Clustal Omega. The 44 alleles encoded a total of 34 different peptides which range between 120 and 289 aa in length. Analysis of the 34 deduced protein sequences revealed three distinct types of σ^N^ (Fig. 2B). In 17/290 (6%) isolates, the putative σ^N^ protein is 289 aa and, similarly to the allele in N. meningitidis 8013, harbors a frameshift mutation and so lacks an RpoN box. In 4/290 (1%) of the isolates, the *rpoN* allele contains stop codons that result in truncated proteins. In the remaining 269 of 290 isolates (93%), the putative σ^N^ protein was as described by Rendon et al. (23), i.e., a protein which lacks a recognizable HTH domain but has an RpoN box in the C terminus. Analysis of the distribution of each of the three types of σ^N^ (frameshift, truncation, or deletion) in the isolates according to *pilE* locus demonstrated that 77/77 (100%) isolates with a class II *pilE* locus and 192/213 (90%) isolates with a class I *pilE* locus harbor an *rpoN* allele that encodes σ^N^ with an RpoN box but lacking the putative HTH region (Fig. 2C). Given the presence of a conserved RpoN box, we hypothesized that this atypical form of σ^N^ could retain binding to the −24 element and contribute to class II *pilE* transcription from the σ^N^-dependent promoter.

σ^N^-dependent promoters are usually preceded by one or more upstream activator sequences (UAS) situated 80 to 150 bp upstream of the −12/−24 sequence (38, 39). These sequences bind bacterial enhancer binding proteins (bEBPs), which are activated in response to certain stimuli and trigger a conformational change within the holoenzyme via ATP hydrolysis, resulting in the transition between closed and open complex formation and transcription initiation (38, 40). Based on homology to sequences in Pseudomonas aeruginosa, a putative UAS has been identified in the promoter region of class I *pilE* in N. gonorrhoeae and N. meningitidis (21, 22). We used the same UAS from P. aeruginosa (TGTGACACTTTTTGACA) to search for homologous sequences in the promoter regions of the 77 isolates with class II *pilE*. Sequences with 9/17 (TcTGACAaaaaacGtCA) or 10/17 (TcTGACAaaaaaTGtCA) nucleotide matches (unmatched nucleotides are lowercase) were identified approximately 112 bp upstream of the −12/−24 sequence in 50 and 24 of the genomes, respectively, but none of the isolates had an exact match to either the P. aeruginosa sequence or the putative UAS from N. meningitidis or N. gonorrhoeae (not shown).

### *rpoN* is expressed in N. meningitidis, but no protein is detected and it is not required for pilin transcription.

Next, we examined whether σ^N^ can be detected in class II pilin-expressing N. meningitidis by Western blotting. Using cc-11 strain S4, which expresses class II pilin, a mutant was constructed in which the endogenous copy of *rpoN* was replaced with the *rpoN* ORF in frame with a sequence encoding a C-terminal histidine tag. The presence of σ^N^ in whole-cell lysates from bacteria grown overnight on solid medium was then assessed by Western blotting using an anti-His antibody. No protein of the predicted size (34 kDa) was present in extracts from S4*rpoN*_His, indicating that, under the conditions tested, σ^N^ could not be detected in N. meningitidis (data not shown).

**FIG 3:**
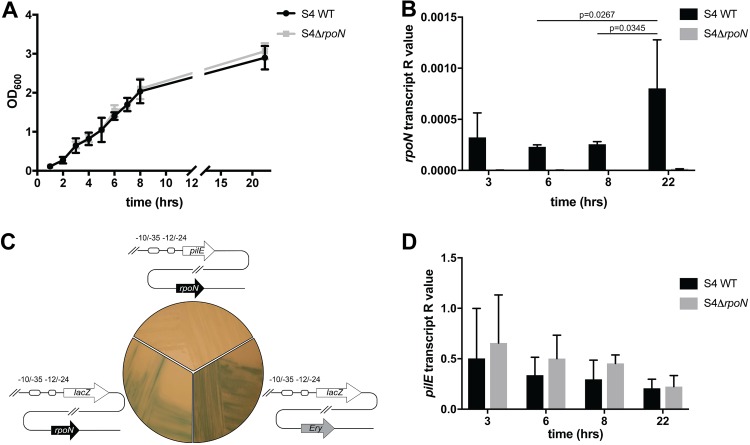
Effect of *rpoN* deletion on *pilE* levels in N. meningitidis. (A) Growth of N. meningitidis WT S4 and S4Δ*rpoN* in liquid medium at 37°C. (B) *rpoN* expression was measured using qRT-PCR at different time points as indicated. There was a significant difference between *rpoN* transcript levels in WT S4 at 22 h and 8 h (*P* = 0.0345) and at 22 h and 6 h, (*P* = 0.0267) (two-way analysis of variance [ANOVA]). No transcript was detected in S4Δ*rpoN*. (C) WT S4, S4ϕP_pilE_-*lacZ*, and S4Δ*rpoN*ϕP_pilE_-*lacZ* were grown on medium containing X-Gal. Blue colonies indicate expression of β-galactosidase and therefore transcription from the *pilE* promoter. Colonies appeared blue even in the absence of *rpoN*. (D) *pilE* mRNA levels at different time points during the growth of WT S4 and S4Δ*rpoN. pilE* mRNA is present throughout bacterial growth, and no significant difference in *pilE* expression was detected, either at different time points or in the absence of *rpoN*. The amount of transcript is presented as an *R* value and normalized to tmRNA. Histograms show pooled data from three independent experiments. Error bars show SD.

We therefore determined whether *rpoN* mRNA was detectable in N. meningitidis S4. Using quantitative reverse transcription-PCR (qRT-PCR) we measured *rpoN* transcript levels during growth of wild-type (WT) S4 in liquid medium at 37°C. RNA from strain S4 Δ*rpoN*, in which the entire *rpoN* coding sequence was replaced with an antibiotic resistance cassette, was used as a negative control for nonspecific amplification. Transcript levels were measured relative to those of transfer-messenger RNA (tmRNA). As shown in Fig. 3A, growth of wild-type S4 was similar to that of S4 Δ*rpoN*, indicating that the absence of *rpoN* does not impact bacterial fitness. *rpoN* transcript was detected throughout growth, albeit at low levels relative to those of the control tmRNA (Fig. 3B). *rpoN* transcript varied according to the growth phase, with highest levels detected during stationary phase (22 h).

To determine whether *rpoN* is required for *pilE* transcription, we next constructed reporter strains in which the class II *pilE* ORF was replaced by *lacZ* at the endogenous *pilE* locus in strains S4 and S4Δ*rpoN* (Fig. 3C). The resulting strains, S4ϕP_pilE_-*lacZ* and S4Δ*rpoN*ϕP_pilE_-*lacZ*, were plated onto solid medium containing 5-bromo-4-chloro-3-indolyl-β-d-galactoside (X-Gal), and the detection of blue colonies was used to qualitatively assess whether σ^N^ is required for *pilE* expression. After overnight growth, blue colonies were detected for both S4ϕP_pilE_-*lacZ* and S4Δ*rpoN*ϕP_pilE_-*lacZ*, indicating that *rpoN* is not required for *pilE* expression. Finally we analyzed *pilE* transcript levels in wild-type S4 and in S4Δ*rpoN. pilE* mRNA was measured by qRT-PCR and expression levels normalized to those of tmRNA. As shown in Fig. 3D, *pilE* was expressed at similar levels throughout growth in the wild-type S4 and S4Δ*rpoN*, demonstrating that σ^N^ is not required for class II pilin expression in S4 under the conditions tested.

### Induced expression of σ^N^ does not impact pilin protein levels in N. meningitidis.

**FIG 4:**
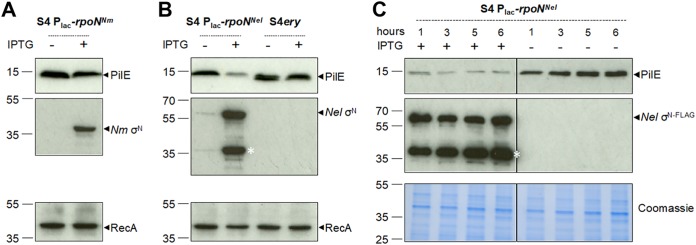
Effect of *rpoN* overexpression on PilE levels in N. meningitidis. (A) S4P_lac_-*rpoN*^Nm^ was grown overnight on solid medium in the presence or absence of IPTG to induce σ^N^ expression. Pilin expression was detected by Western blotting with an antipeptide antibody. Expression of σ^N^ was verified using anti-FLAG antibodies. No change in pilin expression was detected in the presence of σ^N^. (B) S4P_lac_-*rpoN*^Nel^ and control strain S4 *ery* were grown overnight on solid medium with or without IPTG. N. elongata σ^N^ was detected using anti-FLAG antibodies. Expression of σ^N^ from N. elongata in S4 led to a reduction in pilin expression, while no reduction was observed in the control strain. The asterisk indicates a degradation product of Nel σ^N-FLAG^. (C) Similarly, a reduced level of pilin was detected in S4P_lac_-*rpoN*^Nel^ grown in liquid medium in the presence of IPTG for 1, 3, 5, and 6 h compared to that without the inducer. Expression of RecA or Coomassie blue staining of extracts was used as a control. Numbers to the left indicate the positions of molecular weight markers (kilodaltons).

We considered the possibility that the absence of detectable σ^N^ in wild-type S4 could account for the observed lack of any effect of the deletion of *rpoN* on class II *pilE* transcript levels. Therefore, we examined the impact of inducing *rpoN* expression on pilin levels. We constructed N. meningitidis S4 harboring *rpoN* with an in-frame FLAG tag at an ectopic locus (41) under the control of an isopropyl-β-d-thiogalactopyranoside (IPTG)-inducible promoter (S4P_lac_-*rpoN*^Nm^). In parallel, we generated a strain (S4P_lac_-*rpoN*^Nel^) with IPTG-inducible N. elongata *rpoN*, which activates *pilE* transcription in N. elongata by binding the −12/−24 sequence and requires the activator protein Npa (23), and a control strain, S4 *ery*, which lacks the σ^N^-coding sequence at the ectopic locus. Pilin levels in S4P_lac_-*rpoN*^Nm^, S4P_lac_-*rpoN*^Nel^, and S4 *ery* were analyzed by Western blotting of cell lysates collected after overnight growth on solid medium with or without 1 mM IPTG to induce *rpoN* expression; the expression of σ^N^ was confirmed with anti-FLAG antibody. There was no detectable difference in pilin levels in S4P_lac_-*rpoN*^Nm^ in the presence or absence of inducer (Fig. 4A), demonstrating that expression of *rpoN* does not affect class II pilin levels. We therefore conclude that meningococcal σ^N^ is unlikely to bind to the −12/−24 sequences in the *pilE* promoter to activate transcription of class II *pilE*.

Interestingly, the expression of N. elongata *rpoN* in N. meningitidis led to a substantial reduction in pilin expression (Fig. 4B), and this was also observed during growth in liquid medium (Fig. 4C). One possible explanation is that N. elongata σ^N^ can engage with the −12/−24 promoter upstream of class II *pilE* but, in the absence of additional N. elongata specific factors, transcription does not initiate, and the bound sigma factor instead blocks transcription from adjacent promoters. This phenomenon has been described in E. coli, in which an excess of σ^N^ can reduce RNA synthesis by σ^70^ RNAP when the σ^N^ binding site overlaps the region normally occupied by σ^70^ RNAP (42). Furthermore, our finding is consistent with analysis of gonococcal *pilE* promoters in E. coli, which has demonstrated that σ^N^ can reduce levels of transcription of P*pilE-lacZ* or P*pilE-cat* fusions, likely by steric hindrance at the overlapping σ^70^-dependent promoter (43, 44). Analysis of the relative positions of the –10/−35 promoter and the σ^N^ binding site upstream of class II *pilE* suggests that they are sufficiently close for competition to occur (42). Taken together, our data indicate that σ^N^ in N. meningitidis is nonfunctional or does not function as a canonical σ^N^ and that class II *pilE* transcription does not initiate from the −12/−24 promoter.

### Class II *pilE* transcription initiates from the σ^70^-dependent promoter.

**FIG 5:**
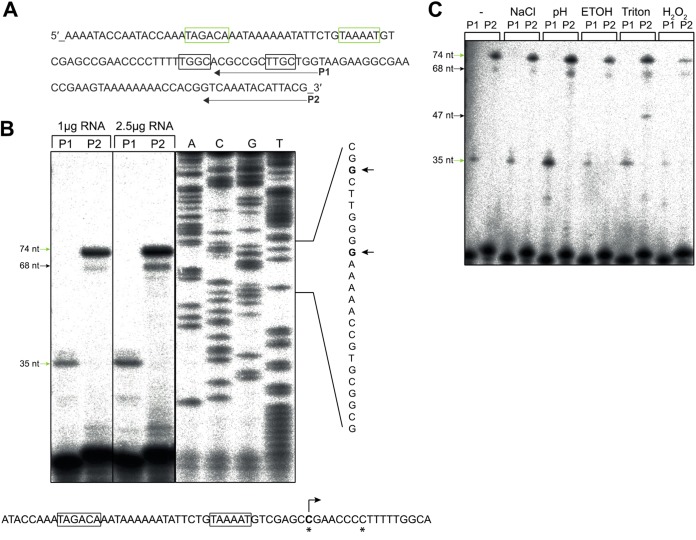
Identification of the class II *pilE* transcriptional start site. (A) Sequence of the region upstream of class II *pilE* in S4, comprising the putative −10/−35 and −12/−24 promoter elements (boxed). The position where primers P1 and P2 bind is indicated. (B) Primer extension was performed using 1 μg or 2.5 μg of RNA prepared from N. meningitidis S4 grown at 37°C in liquid BHI to mid-exponential phase. Products of approximately 35 nt (with primer P1) and 74 nt (with primer P2) were detected, corresponding to transcripts initiating from the −10/−35 promoter. An additional product of ∼68 nt was observed with P2. No product corresponding to transcript initiating from the −12/−24 sequence (∼38 nt) was detected. Lanes A, C, G, and T show sequences determined using a PCR product and primer P2. The transcription start sites (TSS) are indicated by arrows and highlighted by asterisks in the promoter sequence. (C) Primer extension of class II *pilE* transcript in bacteria subjected to different stresses as indicated. No difference in class II *pilE* TSS was observed. An additional extension product (∼47 nt) was detected with the P2 primer in Triton-treated samples. The experiment was performed twice using independent biological replicates.

Next, we mapped the class II *pilE* transcriptional start site (TSS). Total RNA was prepared from N. meningitidis S4 grown in liquid brain heart infusion (BHI) at 37°C to mid-exponential phase, and primer extension was performed using primers designed to capture transcripts generated from both the −10/−35 and the −12/−24 promoters (primer P2) or from only the −10/−35 promoter (primer P1) (Fig. 5A). Extension conditions were such that any TSS within nucleotide (nt) −70 to −250 of the *pilE* start codon would be detected. Totals of 1 μg and 2.5 μg of RNA were used to generate cDNA with radiolabeled P1 and P2. Extension with primer P1 generated a product of ∼35 nt, corresponding to a transcript initiated from the −10/−35 sequence. Similarly, an ∼74-nt product was generated with P2, which corresponds to the same TSS. Interestingly, an ∼68-nt minor product was also generated with P2, which could be an alternative TSS or a degradation product. Importantly, using P2 there was no detectable product of ∼38 nt that would indicate transcription from the −12/−24 promoter, supporting our hypothesis that class II *pilE* transcription initiates from the σ^70^-dependent promoter. Sequencing with primer P2 using a PCR fragment of the upstream region of class II *pilE* as the template identified a cytosine residue located 8 nt downstream of the −10 sequence as the TSS of class II *pilE* (Fig. 5B).

### Class II *pilE* transcript initiation is not affected by stress conditions.

σ factors are often associated with responses to a particular stress, and the level of a given σ factor increases rapidly following an appropriate stimulus (45). σ^N^ was originally identified in the response to nitrogen starvation but has since been implicated in responses to diverse stimuli mediated by specific bEBPs (26). Therefore, to determine whether *pilE* promoter activity changes when meningococci are subjected to different stress conditions, we performed primer extension on RNA from S4 exposed to salt (5 M NaCl), acid (HCl, pH 2.5), oxidative (0.15% H_2_O_2_ ), or envelope (5% ethanol or 5% Triton) stress for 10 min. N. meningitidis S4 grown to the same optical density at 600 nm (OD_600_) but not exposed to any stress was used as a control. As previously, we detected products corresponding to transcript initiation from the −10/−35 promoter under all conditions as well as the ∼68-nt product and a smaller ∼47-nt product with the primer P2 from bacteria exposed to Triton. However, none of the stress conditions led to the appearance of an ∼38-nt product with P2, indicating that the −10/−35 promoter is responsible for class II *pilE* transcription even under different environmental conditions.

### Modulating levels of σ^E^ and σ^H^ does not impact class II pilin expression.

In bacteria, −10/−35 promoter sequences are able to recruit different σ factors belonging to the σ^70^ family (46, 47). For instance, the −10/−35 sequence upstream of *rpoD* in E. coli can bind σ^D^, σ^E^, and σ^S^ (48). Based on sequence similarity to orthologues in E. coli, three σ^70^ factors (σ^D^, σ^H^, and σ^E^) have been identified in N. meningitidis. σ^D^ (encoded by NEIS1466) is considered the “housekeeping” σ factor. In most bacteria this σ factor is essential and is responsible for the transcription of the majority of genes and essential pathways throughout growth (49). Based on the analysis of essential genes in N. meningitidis 8013 and N. gonorrhoeae FA1090, *rpoD* is also essential in pathogenic *Neisseria* species (50, 51). The regulon of σ^H^ (encoded by NEIS0663) and its role in heat shock adaptation and regulation of genes involved in cell adhesion in N. gonorrhoeae has been characterized (30, 52), but the σ^H^ regulon in N. meningitidis has not been described. Finally, σ^E^ (NEIS2123) regulates 11 genes in N. meningitidis (28). The regulon does not include *pilE*, although the analysis was performed in a class I *pilE*-expressing strain. We hypothesized that if the *pilE* −10/−35 promoter binds different σ^70^ members, changing the levels of the sigma factors relative to the level of σ^D^ may impact pilin expression. Therefore, we constructed N. meningitidis mutants lacking or overexpressing σ^H^ or σ^E^ and examined pilin levels in these backgrounds.

**FIG 6:**
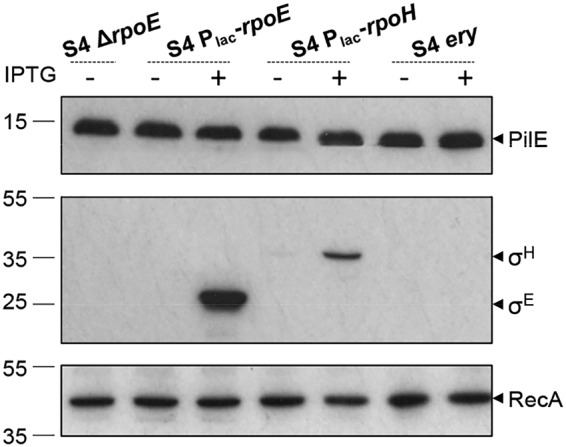
Pilin levels are unaffected by altering levels of σ^H^ or σ^E^. Western blot analysis of pilin levels in whole-cell lysates collected from bacteria lacking σ^E^ (S4 Δ*rpoE*) or with inducible expression of σ^H^ or σ^E^ (S4 P_lac_-*rpoH* and S4 P_lac_-*rpoE*), or from a control strain (S4 *ery*), on solid medium at 37°C is shown. Expression of IPTG-induced σ^E^ and σ^H^ was detected using anti-FLAG antibody. No change in pilin levels was observed when σ factors were deleted or induced. RecA was used as a control, and the blot is a representative from two independent experiments. Numbers to the leftls indicate the positions of molecular weight markers (kilodaltons).

Consistent with σ^H^ being essential in N. gonorrhoeae and N. meningitidis (30, 51), our attempts to construct S4Δ*rpoH* were unsuccessful. Therefore, we generated strain S4P_lac_-*rpoH*, in which σ^H^ expression is inducible by IPTG and detectable due to the presence of a C-terminal FLAG tag. S4P_lac_-*rpoH* was grown overnight on solid medium with or without IPTG, and pilin levels were analyzed by Western blotting. There was no difference in pilin expression in the presence or absence of IPTG (Fig. 6).

As meningococcal σ^E^ is not essential (28), we generated S4Δ*rpoE* by replacing the *rpoE* ORF with a kanamycin resistance cassette, as well as strain S4P_lac_-*rpoE* overexpressing FLAG-tagged σ^E^. Western blot analysis revealed that pilin levels remained unaltered in the absence or induced expression of σ^E^ (Fig. 6). This is consistent with *pilE* not being part of the σ^E^ regulon and indicates that σ^E^ is not essential for pilin production. Since expression and/or deletion of *rpoH* and *rpoE* does not affect class II pilin levels, we conclude that class II *pilE* transcription is initiated from the −10/−35 promoter by σ^D^.

## DISCUSSION

Among the factors that allow bacteria to colonize and infect the human host, Tfp play a key role by meditating adhesion, signaling, and interbacterial attachment (7, 8, 53). Alterations to Tfp, through either changes in sequence, posttranslational modification, or changes in expression, can have important consequences for bacterial behavior. For example, the absence of Tfp significantly reduces the ability of bacteria to attach to host cells (54–56), while modification of the pilin sequence, glycosylation, or phospho-modification can impact bacterial adhesion and aggregation and may contribute to dissemination within or between hosts (5, 57, 58). Therefore, understanding the mechanisms by which Tfp expression and function are modulated is important for understanding pathogenesis and may provide insights into novel ways to overcome meningococcal immune evasion strategies.

Two distinct classes of pilin have been identified in pathogenic *Neisseria* (11, 12). Class I pilins can be altered at high frequency through gene conversion or phase variation (16, 59–62), while class II pilins are invariant. As described in previous studies, the meningococcal class II *pilE* locus is distinguishable from the class I *pilE* locus based on the encoded pilin sequence, the flanking genes, and the presence of sequences required for antigenic variation (13). Our current work confirms that class I and class II *pilE* loci also have different promoter sequences, consistent with previous observations (12). Mirroring the very low nucleotide diversity of class II pilin-coding sequences (13, 19, 20), the class II *pilE* promoter regions were highly conserved and, independent of clonal complex, harbored potential σ^70^ and σ^N^ binding sequences. These findings differ from those of Rendon et al. (23), who defined three types of *pilE* promoter, including one group (represented by FAM18) with σ^N^ but not σ^70^ recognition sequences; instead, we identified putative σ^70^-dependent promoters in all genomes, including FAM18.

Previous studies of *pilE* regulation in pathogenic *Neisseria* spp. showed that transcription of class I *pilE* relies on the σ^70^-dependent rather than the σ^N^-dependent promoter (21, 22, 24). The presence and position of highly conserved, consensus −12/−24 sequences upstream of class II *pilE*, as well as an *rpoN* gene encoding a sigma factor with a conserved −24 binding region, led us to investigate the role of the σ^N^-dependent promoter in class II *pilE* transcription. Based on structure-function studies in other organisms, we considered the possibility that this atypical form of σ^N^ still activates transcription despite lacking the −12 binding HTH domain. For example, mutants which do not effectively recognize the −12 sequence can still produce RNA *in vitro* in an activator-independent manner known as σ^N^ bypass transcription (63). Consistent with this, no GAFTGA-containing bEBPs have been identified in N. meningitidis (64), and Npa, the activator for σ^N^-dependent *pilE* transcription in N. elongata, is truncated in N. meningitidis (23, 65). Furthermore, in E. coli σ^N^ bypass transcription can occur at −12/−24 promoters with a noncanonical −12 element (42). However, our findings indicate that the −12/−24 promoter and σ^N^ do not contribute to class II *pilE* expression; we were unable to detect any σ^N^ protein expression, and neither deletion nor overexpression of *rpoN* had any significant effect on class II *pilE* transcript or protein levels. Furthermore, mapping the TSS of class II *pilE* demonstrated that the σ^70^-dependent promoter is responsible for class II *pilE* expression, even under a variety of stress conditions, some of which are known to induce σ^N^-dependent transcription in E. coli (66). Collectively, our results demonstrate that class II *pilE* transcription is independent of σ^N^ and initiates from the −10/−35 promoter.

An important observation that emerged from our analysis is that in a variety of N. meningitidis mutants lacking sigma factors, as well in the presence of stress signals, *pilE* transcript and/or protein expression remained relatively stable. Considering the importance of Tfp for bacterium-bacterium interactions, competence, and adhesion to host cells, it is likely that class II *pilE* is constitutively expressed to ensure that the capacity to synthesize pilin is maintained. Consistent with this, changing levels of σ^E^, σ^H^, or σ^N^ had no detectable impact on pilin levels. Although we cannot rule out that σ^H^ or σ^E^ may contribute to the expression of class II *pilE* under specific conditions, we found no evidence of σ^N^ expression or activity.

Of note, we found alleles which encode the same σ^N^ mutations or deletions in strains with either a class I or class II *pilE* locus, suggesting that the loss of functional *rpoN* occurred independently of the evolution of the distinct *pilE* loci. Interestingly, the commensal species Neisseria polysaccharea and Neisseria lactamica have class II *pilE* loci that are very closely related to the meningococcal class II *pilE* locus, and in our previous work we proposed that class II pilin-expressing meningococci may have either evolved from a common ancestor or acquired the class II *pilE* gene from these species (13). However, the *rpoN* gene in these nonpathogenic species does not harbor mutations and deletions and potentially encodes a functional σ^N^ (23). Therefore, our findings suggest that loss of functional σ^N^ is beneficial for the pathogenic *Neisseria* spp. and are consistent with the idea that a switch in *pilE* regulation from σ^N^-dependent to σ^D^-dependent control occurred during evolution and divergence of pathogens from commensals (23, 25). As σ^N^-dependent transcription is usually tightly regulated by bEBPs which respond only to specific stimuli, loss of σ^N^-dependent transcription of *pilE* might therefore be an evolutionary pathway to achieve constitutive σ^D^-dependent expression from the −10/−35 promoter. In this way, transcription of the gene encoding the major component of Tfp is maintained, consistent with the significant contribution of this important organelle at multiple stages of colonization and pathogenesis.

## MATERIALS AND METHODS

### Bacterial strains and growth conditions.

The bacterial strains used in this study are listed in Table S2 in the supplemental material. E. coli was grown at 37°C, supplemented with antibiotics where appropriate, either on Luria-Bertani (LB) agar or in 5 ml LB liquid with shaking at 180 rpm. N. meningitidis was grown at the desired temperature in the presence of 5% CO_2_ on brain heart infusion (BHI) (Oxoid) agar supplemented with 0.1% (wt/vol) starch, 5% (wt/vol) heat-denatured horse blood, and antibiotics as appropriate or in BHI liquid. To inoculate liquid cultures, N. meningitidis was grown overnight on BHI agar. A loop of bacteria was harvested from plates and resuspended in 600 μl of phosphate-buffered saline (PBS), and DNA was quantified in lysis buffer (P2 buffer; Qiagen) by measuring the OD_260_. A total of 10^9^ CFU was used to inoculate 25 ml of BHI in a 125-ml conical flask (Corning) and then incubated at appropriate temperatures with shaking at 180 rpm. The OD_600_ of cultures was used to monitor growth. Antibiotics were used at the following concentrations: kanamycin, 50 μg/ml and 75 μg/ml for E. coli and N. meningitidis, respectively; erythromycin, 2 μg/ml; and carbenicillin, 100 μg/ml.

### Bioinformatic analysis.

The 290 isolates whose genomes were used in this study comprise 288 isolates described in our previous work (13) and two additional isolates: S4 and FAM18 (class II *pilE*). Among the 288 isolates, 56 belong to the collection of 107 meningococcal isolates originally used for multilocus sequence type (MLST) development in *Neisseria* (3). The remaining 232 are disease-associated strains isolated during the 2010–2011 epidemiological year from patients in England, Wales, and Northern Ireland and whose genomes are publicly available through the Meningitis Research Foundation genome library (MRF-MGL) on PubMLST (https://pubmlst.org/neisseria/) (67). These isolates were chosen for *pilE* promoter and *rpoN* analysis based on our previous work where we identified them as having either a class I or a class II *pilE* locus (13). BLASTn analysis of genomes in the PubMLST *Neisseria* database was performed to identify *pilE* promoter and *rpoN* sequences. For *pilE* promoter analysis, up to 500 nt of sequence flanking the coding DNA sequence (CDS) was analyzed, and annotation was performed manually based on published *pilE* promoters (12, 21, 32) and bacterial consensus promoter sequences (33). Nucleotide sequences were aligned using Clustal Omega (https://www.ebi.ac.uk/Tools/msa/clustalo/) (68). Percent identity was determined using the percent identity matrix within Clustal Omega. *rpoN* (NEIS0212) of each isolate used in our study was identified by BLAST, results were manually inspected to define coding sequences, and alleles were assigned using PubMLST. Sequences were translated using EMBOSS Transeq (https://www.ebi.ac.uk/Tools/st/emboss_transeq/). N. meningitidis σ^N^ putative domains were identified based on alignment with E. coli σ^N^ using the EMBOSS Water pairwise sequence alignment tool with the default settings (https://www.ebi.ac.uk/Tools/psa/emboss_water/). SnapGene was used to construct plasmid maps and for automatic annotation of features and ORFs in whole-genome sequences.

### Mutant construction.

The primers used in this study are listed in Table S3 in the supplemental material. N. meningitidis strains lacking σ factors were constructed by gene replacement. First, DNA fragments corresponding to 479 bp upstream and 712 bp downstream of the N. meningitidis S4*rpoN* ORF (NEIS0212, homologue of FAM18 NMC_RS01150) and a kanamycin resistance cassette were amplified by PCR using primers ML20/21, ML22/23, and ML24/25, respectively. The upstream and downstream fragments were cloned into pGEM-T Easy flanking a kanamycin resistance cassette, resulting in pGEM-T Easy Δ*rpoN*. This plasmid was digested with BamHI and NotI, and the Δ*rpoN* fragment was purified by gel extraction prior to transformation into S4. Plasmid pUC19Δ*rpoE* was generated by amplifying 483-bp and 459-bp fragments corresponding to regions upstream and downstream of the N. meningitidis *rpoE* (NEIS2123, homologue of FAM18 NMC_RS11230) using primers ML284/ML285 and ML288/ML289, respectively. A kanamycin resistance cassette was amplified using primers ML286/ML287, and the three fragments were ligated into pUC19 using the NEB Builder HiFi DNA assembly kit (New England Biolabs). The construct for replacing *rpoE* with the kanamycin resistance gene was amplified from pUC19Δ*rpoE* by PCR and then purified and used for transformation into S4.

To generate S4 and S4Δ*rpoN* harboring ϕP_pilE_-*lacZ* at the native *pilE* locus, first *rpoN* was replaced with *ermC*, encoding erythromycin resistance. Briefly, regions upstream (485 bp) and downstream (886 bp) of the *rpoN* start and stop codons, respectively, were amplified from existing mutant S4Δ*rpoN* (Kan^r^) genomic DNA (gDNA) using primers ML20/ML46 and ML25/ML52, and overlap PCR was used to clone these fragments 5′ and 3′ of an erythromycin resistance gene, respectively. The product was cloned into pCR2.1TOPO, resulting in pCR2.1TOPOΔ*rpoN*, which was then used as a template to amplify the deletion construct for transformation, generating S4Δ*rpoN* (Ery^r^). Subsequently, the *pilE* ORF was replaced with *lacZ* as follows. A 673-bp region immediately 5′ of the *pilE* ORF was amplified from S4 gDNA using ML37/ML34. A fragment corresponding to the region 3′ of the *pilE* ORF with a kanamycin resistance marker inserted 81 bp downstream of the *pilE* stop codon was amplified from strain S4_*pilE*kanR (R. Exley, unpublished data) using ML30/ML31. The two fragments were cloned into pCR2.1TOPO flanking the *lacZ* gene, which was amplified from pRS415 (69) using primers ML32/ML33. The resulting plasmid, pCR2.1TOPOϕP_pilE_-*lacZ*, was used as a template to amplify DNA for transformation of wild-type S4 or S4Δ*rpoN*.

N. meningitidis strains overexpressing σ factors were constructed by inserting the relevant ORF at the *iga-trpB* intergenic locus (41) of N. meningitidis S4, under the control of an IPTG inducible promoter. For this purpose, pNMC2 was generated. First, a fragment comprising part of the *iga* gene and terminator was amplified from S4 gDNA using primers ML151 and ML149. Next, a fragment comprising *ermC*, the *lac* regulatory region, and a multiple-cloning site (MCS) was amplified from pGCC4 (a gift from Hank Seifert) (Addgene plasmid 37058; http://n2t.net/addgene:37058; RRID:Addgene_37058) using primers ML148/ML150. The two fragments were fused by overlap PCR using primers ML148 and ML151. The resulting PCR product was then combined using Gibson assembly with a fragment amplified from plasmid pNCC1 (70) with primers ML152 and ML153 (providing the origin of replication and kanamycin selection marker) and a fragment comprising the 3′ end of the *trpB* gene amplified from S4 gDNA using primers ML146 and ML147. Genes encoding C-terminally tagged sigma factors were amplified as follows. The *rpoN*, *rpoE*, and *rpoH* (NEIS0663, homologue of FAM18 NMC_RS03540) genes were amplified from S4 gDNA using primers ML154/ML155, ML280/ML282, and ML184/ML188, respectively. The PCR products were ligated into HindIII-digested pNMC2 using the NEB Builder HiFi DNA assembly kit to generate pNMC2*rpoN*^Nm^, pNMC2*rpoE*, and pNMC2*rpoH*. pNMC2*rpoN*^Nel^ was generated using Gibson assembly with a fragment amplified from pNMC2*rpoN*^Nm^ using ML172/ML174 and a fragment comprising *rpoN* from N. elongata (NELON_RS02880) amplified using gDNA from strain 29315 and primers ML173/ML175. Plasmids were linearized by digestion with NcoI to ensure double-crossover recombination and prevent integration of the entire plasmid and were used to transform S4. DNA from single transformants selected on erythromycin was analyzed by PCR, and gDNA from PCR-positive colonies was backcrossed into the parental strain. Backcrossed colonies were pooled, and gDNA from the pooled stock was checked by PCR and sequencing of the region of interest.

### RNA isolation.

N. meningitidis was grown at 37°C in liquid BHI, and at the indicated times a volume equivalent to an OD_600_ of ≈8 was centrifuged at 4,000 rpm for 10 min. The supernatant was removed, and total RNA was isolated from pellets using TRIzol extraction (Thermo Fisher). Briefly, cell pellets were resuspended in 2 ml of TRIzol and incubated at room temperature for 5 min before adding 200 μl of chloroform. Samples were shaken by hand for 15 s, incubated at room temperature for 2 to 3 min, and then centrifuged at 12,000 × *g* for 15 min at 4°C, and the aqueous phase was transferred to a fresh tube. RNA was precipitated by adding 500 μl isopropanol, followed by centrifugation at 20,000 × *g* for 30 min at 4°C. RNA pellets were washed with 75% ethanol and then air dried and resuspended in diethylpyrocarbonate (DEPC)-treated water. Following DNase treatment for 2 h at 37°C, RNA was extracted with phenol-chloroform (5:1). The aqueous phase was reextracted with DEPC-treated water and chloroform-isoamyl alcohol. Samples were vortexed and centrifuged at 20,000 × *g* for 5 min at 4°C. The aqueous phase was transferred to a tube containing 33 μl of 3 M Na acetate (pH 4.5) and 812.5 μl of 99.5% ethanol, and RNA was precipitated over 2 h or overnight at –20°C. Following centrifugation at 20,000 × *g* for 30 min at 4°C, RNA pellets were washed with 75% ethanol, centrifuged again for 10 min at 20,000 × *g*, air dried, and resuspended in 200 μl DEPC-treated water.

### qRT-PCR.

All primers used in quantitative reverse transcription-PCR **(**qRT-PCR) are listed in Table S3. RNA (2.5 μg) was used to synthesize cDNA with primers specific for the target gene containing a tag sequence (CCGTCTAGCTCTCTCTAATCG) which is not present in the N. meningitidis genome. RNA was reverse transcribed using reverse transcriptase III polymerase (Invitrogen) and treated with RNase H for 20 min at 37°C. cDNA was purified using the Wizard gel PCR purification kit (Promega). qRT-PCR was performed using Power SYBR green PCR master mix (Applied Biosystems). Primer efficiency was evaluated using serial dilutions of gDNA to generate a standard curve, and the slope of the standard curve for each primer pair was calculated. StepONEPlus real-time PCR software was used to collect qRT-PCR data; the Δ*C_T_* method (where *C_T_* is the threshold cycle) was used to analyze the data (71). Results are presented as the normalized *R* value (where the *R* value is calculated as 2^−^*^CT^*) relative to tmRNA. Experiments were repeated at least three times, each time with triplicate samples. The results shown are means and standard deviations (SD) of three biological replicates; tmRNA was used as a control.

### Primer extension.

Primer extension was carried out using 1 μg and 2.5 μg of RNA isolated from N. meningitidis grown to mid-log phase (OD_600_ ≈ 0.4 to 0.5). Primers (P1 and P2 [Table S3]) were radiolabeled using [γ-^32^P]ATP (PerkinElmer). A mix of radiolabeled primers and RNA was incubated for 3 min at 95°C and rapidly cooled on ice for 4 min. Reverse transcription was performed using Superscript II (Invitrogen) by incubating samples at 45°C for 60 min then stopped by deactivating the enzyme for 10 min at 70°C. Formamide loading dye (10 μl) was added to each reaction mixture, and samples were heated briefly at 95°C. Fifteen microliters of each sample was loaded on a 12% denaturing polyacrylamide sequencing gel, and electrophoresis was performed for 2 to 3 h at 20 to 25 W until bromophenol blue dye reached the bottom of the gel. Products were analyzed using a phosphorimager (FujiFilm). Sanger sequencing was performed using primer P2 and a DNA cycle sequencing kit (Jena Bioscience) with a PCR-amplified fragment of the promoter region of *pilE* from S4 as a template.

### Western blot analysis.

N. meningitidis whole-cell extracts were prepared from bacteria grown overnight at 37°C in the presence of 5% CO_2,_ on solid medium or in liquid with appropriate antibiotics with or without 1 mM IPTG. Suspensions were normalized to an OD_600_ of 1 in 1 ml BHI and then centrifuged for 5 min at 12,000 × *g*, and the pellets were resuspended in an equal volume of sterile water and 2× SDS-PAGE lysis buffer (1 M Tris-HCl [pH 6.8], 10% SDS, 30% glycerol, 1% bromophenol blue) containing 200 mM β-mercaptoethanol. Samples were boiled for 10 min prior to electrophoresis. Proteins were separated on 12% polyacrylamide gels and transferred to nitrocellulose membranes (Hybond-C Extra [Amersham] or Immobilon-P polyvinylidene difluoride [PVDF] [Millipore]) using a Bio-Rad Trans-Blot Turbo system. Membranes were blocked for 1 h at room temperature or overnight at 4°C in PBS–5% milk. Western blotting was performed using anti-FLAG antibody (F1804; Sigma) at 1:5,000 and goat anti-mouse IgG–horseradish peroxidase (HRP) (final dilution, 1:10,000; Dako) or using antipilin (EP11270 antipeptide, 1:10,000) and goat anti-rabbit IgG–HRP (final dilution, 1:10,000; Santa-Cruz). Anti-RecA (1:5,000) (ab63797; Abcam) followed by goat anti-rabbit IgG–HRP (final dilution, 1:10,000; Santa-Cruz) or Coomassie blue staining was used as a loading control. All incubations with antibodies were for 1 h at room temperature in PBS–5% milk containing 0.1% Tween. Membranes were washed three times with PBS–0.1% Tween between incubations. Cross-reacting proteins were visualized using ECL detection reagent (GE Healthcare).

## Supplementary Material

Supplemental file 1

Supplemental file 2
