# Supplementary material for: Contribution of σ70 and σN Factors to Expression of Class II pilE in Neisseria meningitidis
Source: J Bacteriol. 2019 Sep 20;201(20):e00170-19. doi: 10.1128/JB.00170-19 (PMC6755734; doi:10.1128/JB.00170-19)
Supplement: Supplemental file 1 [file JB.00170-19-s0002.pdf]

A

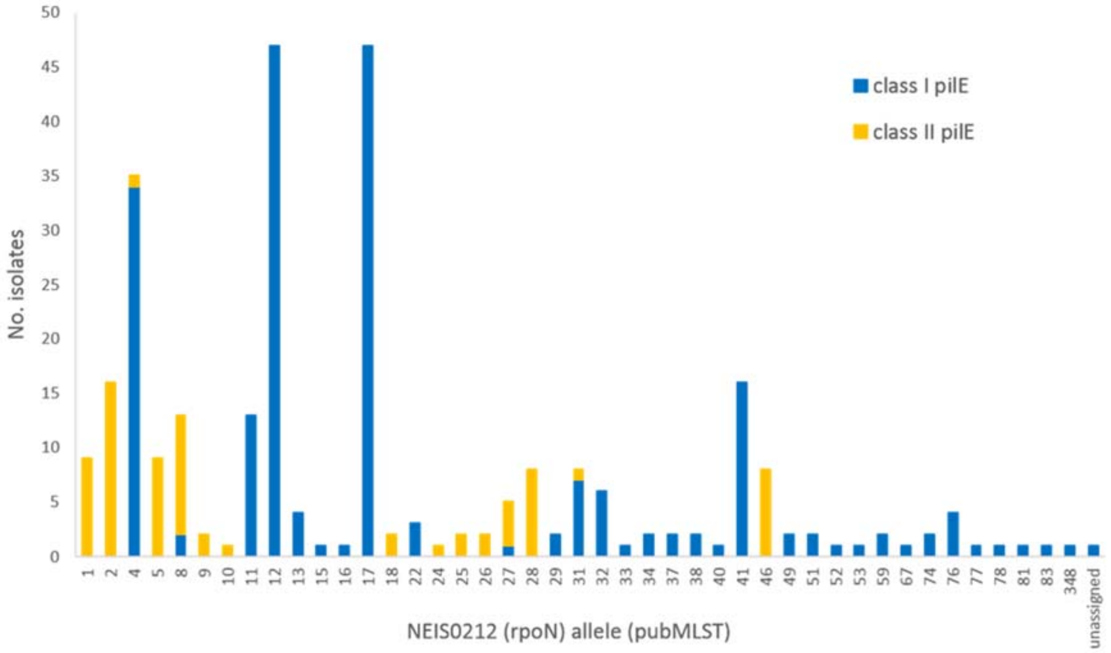

Fig. S1. Prevalence of *rpoN* alleles in meningococcal genomes analysed is this study.

A total of 290 meningococcal genomes were analysed. We identified 44 unique *rpoN* (NEIS0212) alleles among the 290 isolates. Isolates with class I *pilE* are indicated by blue bars, isolates with class II *pilE* locus are indicated by yellow bars. Of the 44 different alleles, 11 are exclusive to isolates with class II *pilE* and 29 are found only in isolates with class I *pilE*. Alleles 4, 8,27 and 31 were found in isolates with class I *pilE* or class II *pilE*.

SUPPLEMENTARY TABLES

Table S2: Bacterial strains used in this study

**Strain**	**Genotype/Description**	**Source**
E. coli		
Dh5α	F^{-} *endA1 glnV44 thi-1 recA1 relA1 gyrA96 deoR nupG purB20* ϕ80d*lacZ*ΔM15 Δ(*lacZYA-argF*)U169, hsdR17($r_K^{-}m_K^{+}$), λ^{-}	Invitrogen
N. meningitidis		
S4	Serogroup C, ST-11 complex/ET-37, class II *pilE*	(1)
S4Δ*rpoN*	*rpoN* replaced with kanamycin or erythromycin resistance marker	This study
S4Δ*rpoE*	*rpoE* replaced with kanamycin resistance marker	This study
S4ϕP_{pilE}*lacZ*	Reporter strain for class II *pilE* promoter activity. S4 with *lacZ* in place of *pilE* ORF at native locus. Kanamycin resistant.	This study
S4Δ*rpoN* ϕP_{pilE}*lacZ*	Reporter strain for class II *pilE* promoter activity. S4Δ*rpoN (erm(C))* with *lacZ* in place of *pilE* ORF at native locus. Kanamycin and erythromycin resistant.	This study
S4P_{lac}-*rpoN*^{Nm}	S4 with gene encoding FLAG-tagged σ^N from *N. meningitidis* strain S4 integrated at *trpB-iga* locus under control of IPTG inducible promoter. Erythromycin resistant.	This study
S4P_{lac}-*rpoN*^{Nel}	S4 with gene encoding FLAG-tagged σ^N from *N. elongata* strain 29315 integrated at *trpB-iga* locus under control of IPTG inducible promoter. Erythromycin resistant.	This study
S4 *ery*	S4 with empty vector integrated at *trpbB-iga* locus. Erythromycin resistant.	This study
S4P_{lac}-*rpoE*	S4 with gene encoding FLAG-tagged σ^E integrated at *trpB-iga* locus under control of IPTG inducible promoter. Erythromycin resistant.	This study
S4P_{lac}*rpoH*	S4 with gene encoding FLAG-tagged σ^H integrated at *trpB-iga* locus under control of IPTG inducible promoter. Erythromycin resistant.	This study

1. **Uria MJ, Zhang Q, Li Y, Chan A, Exley RM, Gollan B, Chan H, Feavers I, Yarwood A, Abad R, Borrow R, Fleck RA, Mulloy B, Vazquez JA, Tang CM.** 2008. A generic mechanism in *Neisseria meningitidis* for enhanced resistance against bactericidal antibodies. J Exp Med **205:**1423-1434.

Table S3: Primers used in this study

Name	Description/Purpose	SEQUENCE 5'-3'
ML20	Forward primer for upstream fragment for *rpoN* deletion in S4	CACGAATTCTTTTCATTCAGCCACAAAT
ML21	Reverse primer for upstream fragment for *rpoN* deletion in S4	CCAGGTACCGATAATGTGGGAGAAATTGT
ML22	Forward primer for kanamycin resistance marker for *rpoN* deletion in S4	CCAGGTACCAATTGTGTCTCAAAATCTCTGA
ML23	Reverse primer for kanamycin resistance marker for *rpoN* deletion in S4	CCAGGTACCCGATGCATGCCAACAGATAA
ML24	Forward primer for downstream fragment for *rpoN* deletion in S4	CCAGGTACCTTGCTGAATAATCTTATAAAGAC
ML25	Reverse primer for downstream fragment for *rpoN* deletion in S4	CCAGGATCCTTTCGGTCATTTCTGATAAAC
ML284	Forward primer for upstream fragment for *rpoE* deletion in S4	GATCCTCTAGAGTCGACCTGCAGGCATGCACTTTTAGACGGCATTTTGGCACTG
ML285	Reverse primer for upstream fragment for *rpoE* deletion in S4	CCCGTTGAATATGGCTCATAGTATCGGGAACAAGGTAAT
ML288	Forward primer for downstream fragment for *rpoE* deletion in S4	GCTCGATGAGTTTTTCTAAAACACCGCACGGGTATTTAC
ML289	Reverse primer for downstream fragment for *rpoE* deletion in S4	CAGGAAACAGCTATGACCATGATTACGCCATGCTGCTTTTGAAGCGTG
ML286	Forward primer for kanamycin fragment for *rpoE* deletion in S4	AATTACCTTGTTCCCGATACTATGAGCCATATTCAACGGG
ML287	Reverse primer for kanamycin fragment for *rpoE* deletion in S4	GTAAATACCCGTGCGGTGTTTTAGAAAAACTCATCGAGCATC
ML46	Reverse primer for upstream fragment for *rpoN* deletion in S4ϕP_{pilE}*lacZ* strains	GGATCCAAGGGCGATAATGTGGGAGAA
ML52	Forward primer for downstream fragment for *rpoN* deletion in S4ϕP_{pilE}*lacZ* strains	TCCCCGGGAAGGGCTCAGAATTGGTTAAT
ML37	Forward primer for upstream (5') fragment for replacement of class II *pilE* ORF with *lacZ* in S4ϕP_{pilE}*lacZ* strains	ATCCCACCAACCCACTTTTCCGC
ML34	Reverse primer for upstream (5') fragment for replacement of class II *pilE* ORF with *lacZ* in S4ϕP_{pilE}*lacZ* strains	TGAATCCGTAATCATGGTCATTTGGATGACTCCTG
ML31	Forward primer for downstream (3') fragment for replacement of class II *pilE* ORF with *lacZ* in S4ϕP_{pilE}*lacZ* strains	TGGTGTCAAAAATAACACCAAATAAGGACA

ML30	Reverse primer for downstream (3') fragment for replacement of class II *pilE* ORF with *lacZ* in S4ϕP_{pilE}*lacZ* strains	TCACGATAGGCGAGGCGCA
ML33	Forward primer amplification of *lacZ* from pRS415 fragment for replacement of class II *pilE* ORF with *lacZ* in S4ϕP_{pilE}*lacZ* strains	CAGGAGTCATCCAAATGACCATGATTACGGATTCA
ML32	Reverse primer amplification of *lacZ* from pRS415 fragment for replacement of class II *pilE* ORF with *lacZ* in S4ϕP_{pilE}*lacZ* strains	TGTCCTTATTTGGTGTTATTTTTGACACCA
ML151	Forward primer for amplification of 3' end of *iga* gene for pNMC2 construction	GAGATTTCGATTCCACCGCCGCCTTATGCAGTCAGCACCAATAC
ML149	Reverse primer for amplification of 3' end of *iga* gene for pNMC2 construction	GAACATGATGAGTGATCGTTAAATTTTGTTAATCCACTATAAAAATGCC
ML148	Forward primer for amplification of *erm*(C) and *lac* regulatory region from pGCC4, for pNMC2 construction	TTATTTTGCTATGAGGGATCCGCTAGCACTAG
ML150	Reverse primer for amplification of *erm*(C) and *lac* regulatory region from pGCC4, for pNMC2 construction	AACAAAATTTAACGATCACTCATCATGTTC
ML152	Forward primer for amplification of plasmid backbone from pNCC1, for pNMC2 construction	AAGGCGGCGGTGGAATCG
ML146	Forward primer for amplification of 3' end of *trpB* gene for pNMC2 construction	CGCGGAATTCTCATGTTTGACAGCTTATAAGCGCGAAGACTTGAACCAC
ML147	Reverse primer for amplification of 3' end of *trpB* gene for pNMC2 construction	CTAGTGCTAGCGGATCCCTCATAGCAAAATAAAATGCCGT
ML153	Reverse primer for amplification of plasmid backbone from pNCC1, for pNMC2 construction	ATAAGCTGTCAAACATGAGAATTCCGC
ML154	Forward primer for amplification of *rpoN* from S4. For cloning into pNMC2	CCCAAGCTTGGAGTAATTTTATGACCTTACTCGGAATAAAGCT
ML155	Reverse primer for amplification of *rpoN* from S4. For cloning into pNMC2. Primer includes sequence for in frame triple FLAG TAG	GTGGTTTAAACTCACTTGTCGTCGTCGTCCTTGTAGTCGATGTCGTGGTCCTTGTAGTCACCGTCGTGGTCCTTGTAGTCTTCTGCGGTTTTGCGTTT
ML173	Forward primer for amplification of *rpoN* from *N. elongata*. For pNMC2*rpoN*^{Nel} construction	TTACGAATTCCCGGATTAATTAAAAGCTTGGAGTAATTTTATGACATTGCTCGGATTAAAACT

ML175	Reverse primer for amplification of *rpoN* from *N. elongata*. For pNMC2*rpoN*^{Nel} construction	CTTGTAGTCACCGTCGTGGTCCTTGTAGTCGATCCTGCGTTGGTGTGC
ML172	Forward primer for amplification of pNMC2 fragment for pNMC2*rpoN*^{Nel} construction.	GACTACAAGGACCACGACGGTGAC
ML174	Reverse primer for amplification of pNMC2 fragment for pNMC2*rpoN*^{Nel} construction.	AAGCTTTTAATTAATCCGGGAA
ML280	Forward primer for amplification of *rpoE* from S4. For cloning into pNMC2	CCATGATTACGAATTCCCGGATTAATTAAAGGAGTAATTTTATGCCGCTACCCGACCT
ML282	Reverse primer for amplification of *rpoE* from S4. For cloning into pNMC2. Primer includes sequence for in frame triple FLAG TAG	TCACTTGTCGTCGTCGTCCTTGTAGTCGATGTCGTGGTCCTTGTAGTCACCGTCGTGGTCCTTGTAGTCCTTCGGGTTTTCTTGGTTGAAC
ML184	Forward primer for amplification of *rpoH* from S4. For cloning into pNMC2	ACCATGATTACGAATTCCCGGATTAATTAAAGGAGTAATTTTATGAATAACGCTTTCGC
ML188	Reverse primer for amplification of *rpoH* from S4. Primer includes sequence for in frame triple FLAG TAG	TCGTTAAATTTTCACTTGTCGTCGTCGTCCTTGTAGTCGATGTCGTGGTCCTTGTAGTCACCGTCGTGGTCCTTGTAGTCAACCGCTTCGGCTTCTTC
ML66	cDNA synthesis for *rpoN*	CCGTCTAGCTCTCTCTAATCGATGCTGTCGAGGGCGTTTCG
ML67	Strand-specific RT-PCR for *rpoN*	CAAGCCCTGACTGCATTGC
ML69	cDNA synthesis for *pilE*	CCGTCTAGCTCTCTCTAATCGCCTTCAACCTTAACCGATGC
ML70	Strand-specific RT-PCR for *pilE*	AAGGTCAAAAATCCGCAGTG
ML71	cDNA synthesis for tmRNA	CCGTCTAGCTCTCTCTAATCGAACCCGGTAGGAAACCAGTC
ML72	Strand-specific RT-PCR for tmRNA	AGTCGCAAACGACGAAACTT
ML68	Strand-specific RT-PCR tag primer	CCGTCTAGCTCTCTCTAATCG
P1	Primer extension to identify class II *pilE* TSS	CCTTCTTACCAGCAAGCGGC
P2	Primer extension to identify class II *pilE* TSS	CGTAATGTATTTGACCGTGG

Underlined overlap sequences that are not present in genomic sequence added for Gibson cloning. FLAG tag is double underlined.
